# Coexistence of genetically modified seed production and organic farming in Chile

**DOI:** 10.1080/21645698.2021.2001242

**Published:** 2022-01-03

**Authors:** M.A. Sánchez, H Campos

**Affiliations:** a ChileBio CropLife, Santiago, Chile; b International Potato Center, Lima, Peru

**Keywords:** GMO, biotechnology, co-existence, agriculture, seed production, organic farming, Chile

## Abstract

The seed industry in Chile has thrived since the implementation of a stringent, voluntarily self-imposed coexistence strategy between genetically modified organisms (GMOs) and non-GMO seed activities. GMO varieties of maize, soybean, and canola represent the vast majority of biotech seeds produced in Chile. Chile’s exports of genetically modified (GM) seeds and organically grown food products (which excludes GM seeds and materials) continue to expand. Organic Chilean farmers predominantly produce and export fruits such as blueberries, wine grapes, and apples. Under normal agricultural conditions, the inadvertent presence of GMOs in non-GMO or organic crops cannot be ruled out. Producers of organic foods are required to implement stringent measures to minimize contact with any non-organic crop, regardless of whether these crops are GM. Only very small amounts of organic maize, soybean, and canola – if any – have been produced in Chile in recent years. Given the characteristics and nature of Chile’s agriculture, the direct impact of the GM seed industry on organic farming in Chile is likely to be negligible. The Chilean experience with coexistence between GM seed and organic industries may inform other countries interested in providing its farmers with alternative agricultural production systems.

## Introduction

Certified organic, conventional [i.e., non-genetically modified organisms (GMOs)], and GMO farming models are all subject to production requirements and must meet certain standards to commercialize their produce. These standards may be not reached in cases in which animal, plant, or soil material and farming supplies, such as fertilizers or pesticides used in other farming models, are inadvertently transferred by wind, water, or human transportation to neighboring farms. In such cases, farmers or companies may experience profit losses or reputational damage.^1–3^ Specifically, if a certified organic operation is found to have used prohibited substances or GMOs, it may face disciplinary measures such as loss of certification and financial penalties. In this context, the debate has focused on strategies and policies to prevent the economic impacts of GMO “contamination” of organic crops, such as the imposition by non-GMO farmers of stringent criteria to determine organic status.^1,4–6^ Although undesirable commingling is occasionally referred to as “contamination,” the consensus term is adventitious presence, which describes the inadvertent presence of GM seeds or other materials in both conventional and organic crops.^7^

In order for a farming model attractive to farmers and remaining economically viable, coexistence approaches have been proposed.^8^ Such approaches encompass policies, measures, and good practices that allow different agricultural production models to thrive within the same region and products of diverse origin to be transported and marketed side by side, while preserving their identity according to purity standards. Further, these approaches are key to safeguarding the freedom of choice afforded to both farmers and consumers, so that they can use or acquire products according to their preferences and beliefs.^9^

Although measures to reduce the likelihood of the adventitious presence of GMOs in organic products are regularly implemented by farmers, eliminating the risk entirely is not possible. Standardized agronomic practices and communications with neighboring farmers are crucial to minimize adventitious presence; suitable coexistence measures during cultivation, harvesting, transportation, storage, and processing will significantly aid in this endeavor.^10^ For instance, pollen barriers, crop rotation, control of volunteer plants, and spatial and/or temporary isolation are coexistence measures that can contribute to reducing the risk of commingling through minimizing or avoiding cross-pollination between sexually compatible species. Likewise, other measures capable of strengthening coexistence strategies include setting thresholds of varietal impurity in the seed source, thorough cleaning of the harvest, of transportation and processing equipment, and of storage facilities, and the implementation of mechanisms to trace food backward from the store to the farm.

Coexistence does not relate to environmental or health safety, nor to agronomic performance; rather, it relates to food production, providing farmers with options, economic issues, fair market access, respect for consumer preferences, and the perceived value of a product, particularly in the case of export markets. Chile currently lacks regulations and guidelines addressing this topic. Globally, Chile has been a major player in GM seed production over the past three decades.^11^ On the other hand, organic farming in Chile is becoming increasingly attractive to both farmers and consumers. Organic exports from Chile reach more than 50 countries, and the cultivated area of some crops has significantly increased in recent times.^12^

In Chile, as in other countries, organic farmers associations have argued that coexistence between GM seed production and both organic farming and organic apiculture is not feasible. This paper aims to assess how different agricultural models, such as organic farming and GM seed production, can coexist in Chile in the current unregulated climate; it also aims to provide insights to facilitate the discussion of future evidence-based policies.

## Chilean Certified Organic Farming

According to official figures from Chile’s Agricultural and Livestock Service (SAG) (http://www.sag.cl/sites/default/files/estadisticas_nacionales_de_produccion_organica_2019.pdf), Chile’s Ministry of Agriculture agency, which is in charge of organic certification, 20,987 cultivated hectares were certified as organic in Chile in 2019. These hectares represent less than 1% of all lands devoted to annual and permanent crops, to permanent and rotation forage pastures, and to fallow in Chile. This organic production primarily comprises fruit species, which account for 69.5% of the total organic cultivated area. The main major organic fruit grown are wine grapes (3,507 ha, representing 2.6% of all hectares devoted to wine grape production in Chile), apples (2,683 ha, 8.3%), and walnuts (236 ha, 0.6%); whereas, the main minor organic fruits are blueberries (3,868 ha, 24.6%) and raspberries (1,222 ha, 38.1%). Chilean organic crops also include pasture crops (1,413 ha); medicinal plants (374 ha); cereals, pseudocereals (quinoa and amaranth), and oilseeds (273 ha); vegetables and legumes (150 ha); and seeds and nurseries (31 ha). Furthermore, a significant land area (92,279 ha) is devoted to organic wild-product collection of various fruits and plant tissues, namely, rosehip, maqui berries, and brambleberries. In addition, 10,123 hives are certified organic,^12^ representing 0.8% of all registered hives in Chile.

Chile’s certified organic production is mainly destined for export markets. In 2019, 86,948 tons were exported with a free on board (FOB) value of USD 274 million, which accounts for 2.7% of all agricultural exports from Chile. When compared to data from 2015, exports have grown by 32% and their value by 27% (FOB USD), respectively.^12^

## Legal Framework

It is worth noting that in order to be officially certified as an organic product in Chile, whether destined for domestic or export markets, several requirements must be met regarding Law 20.089, otherwise referred to as the Chilean Organic Production Standards created by the National System of Organic Certification. This legal instrument allows two certification systems: i) via third-party certification companies, and ii) self-certification through organic farmers associations; both systems are supervised by SAG and comply with identical regulations.

According to this regulation, GMOs and products thereof are banned in organic agricultural practices. Further, the concurrent production of organic and conventional (i.e., non-GMOs) products, is also banned; however, throughout the first 3 years a specific productive unit (i.e., field or farm) has entered the organic system, both types of productions are allowed. There must be a 6-meter buffer zone between organic and conventional productive units to avoid contamination. When measures to control pests are ineffective, organic farmers can use pesticides based on natural active substances; if these are ineffective or unavailable, the use of certain synthetic pesticides is permitted.

Organic apiaries, including hives in transhumance (i.e., beehives being transported across various locations to exploit the seasonal availability of flowering crops) must be located at a minimum distance of 3 km from areas (e.g., urban and industrial centers, landfills) exposed to the use of methods, products, or activities that can affect the organic status of the beehives.

Primary and processed organic products may be labeled as “100% organic,” “organic,” “produced with organic ingredients,” or “contains organic ingredients in less than 70%,” as appropriate. Organic products for export, produced and certified under foreign organic standards, different from the national requirements, must be labeled in accordance with the specific requirements of the country of destination.

Chile’s Ministry of Agriculture has led the implementation and functioning of the Organic Agriculture National Commission. Its main objective is to establish organic farming as a main component of the country’s sustainable agricultural development. Further, it aims at Chile’s recognition abroad for the high quality of its organic products.

## Chilean GM Seed Industry

Owing to its advantageous geographical, climatic, and economic characteristics, Chile has become a leading player in the development of GM crops. Chile is the main exporter of GM seeds from the southern hemisphere and has amassed 30 years of experience producing GM seeds. Further, field testing activities carried out in Chile allow accelerating global breeding programs, thereby serving diverse countries in the northern hemisphere. Both activities are conducted predominantly by multinational companies via their Chilean subdivisions; however, farmers and local and international companies acting as third parties also play a key role as growers or seed tollers.

Over the seasons spanning from 2015/2016 until 2019/2020, GM seeds of maize, soybean, and canola accounted for more than 99.9% of all GM seeds sown in Chile. Throughout the seasons, maize has represented 50–58% of total GM seed hectares, followed by canola (20–32%) and soybean (17–22%).^13^ Among these three key crops, the rate of GMO production has reached significant levels in Chile. For instance, during the 2017/2018 season, GM maize represented 72% of the entire maize seed production, and 100% of soybean seed production was of GM nature. In the 2019/2020 season, GM canola seed accounted for 85% of all canola seed production.^13^

Chile’s GM seed production is grown exclusively for export markets. The seed value exports (FOB) have ranged between USD $68–93 million between the 2015/2016 and 2019/2020 seasons. Furthermore, field testing and research and development activities have represented between USD $21–25 million in services throughout each of those seasons. GM seeds exports reached a peak in season 2012/2013, reaching USD $324.5 million FOB, because of a severe seed production shortage observed in the United States following adverse weather conditions.

**Figure 1.**
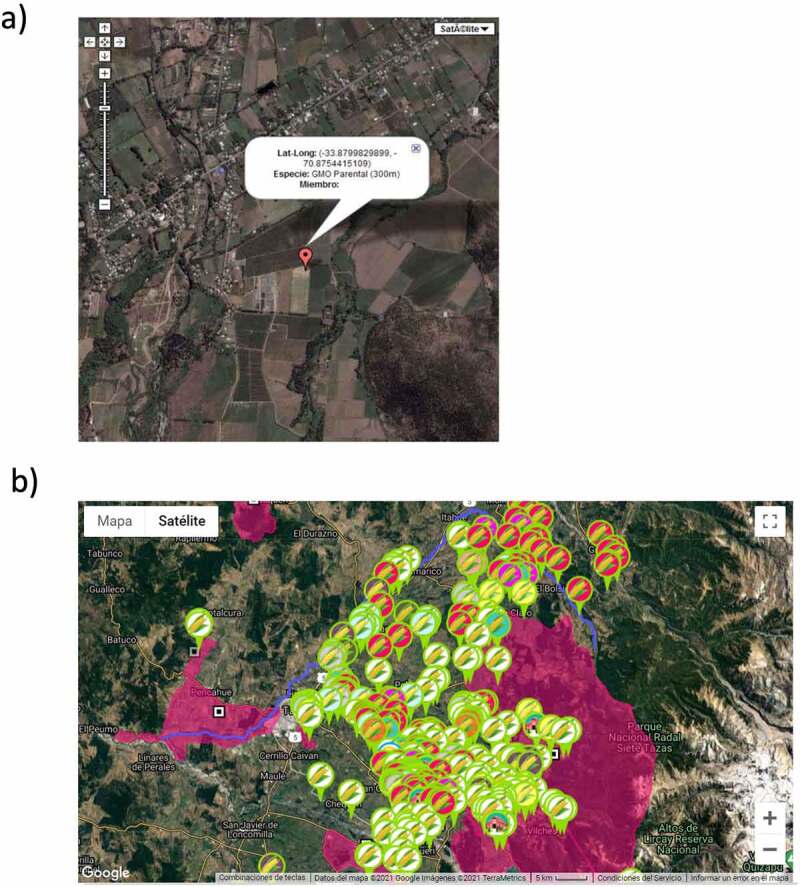
Chile’s Seed trade association (ANPROS) isolation system screenshots. This GPS-based platform enables coexistence between different varieties of non-GMO crops and between GMOs and non-GMOs. a) Georeferenced position of a GMO field; b) Multiple seed corn fields spatially arranged to avoid cross-pollination in order to maintain the reliability and quality of seeds.

Spatial isolation requirements for seed production are crucial for maximizing yield and genetic purity while minimizing economical risk to growers. Therefore, Chile’s seed industry has voluntarily deployed a coexistence strategy between sexually compatible crops. This system has allowed multiple companies to conduct activities using either GM or non-GM seeds. Seed companies use a Global Positioning System (GPS)-based software that is operated and supervised by the national seed trade association (ANPROS). This system, launched in 1999, accurately registers farm positions and identifies the radius in which outcrosses may eventually occur. Further, an isolation handbook has been developed that defines the minimum isolation distances and registration deadlines for each seed-producing farm. Additionally, real-time maps are produced showing all seed production fields of diverse crops (Fig. 1). This allows the stringent monitoring of seed fields of all companies and growers, and also establishes corresponding spatial isolations to avoid non-desired outcrosses, thereby contributing to assure varietal purity. This system has been used to produce seeds safely in areas or regions of interest for many vegetable seeds as well as corn, canola, and sunflower seeds among others. Since vandalism and destruction of GMO field trials by environmental activists has become a relevant phenomenon with important consequences in different countries,^14^ to prevent such kind of acts against farmers and GMO fields, the locations keep in this system is not public information. It is important to point out that never has been reported or known any case of vandalism against GMO fields in Chile.

In addition to the GPS based software, the Excellence Through Stewardship (ETS) program (http://excellencethroughstewardship.org) has successfully enabled the adoption of quality management systems for GMO seeds. Members must renew their audited status every three years. Currently, nine seed companies operating in Chile have successfully completed ETS audit requirements and have in place stewardship programs and quality management systems consistent with this initiative. This has contributed to avoid trade disruptions while producing and exporting seeds for GMO and GMO-free markets.

## Regulatory Framework

The production of GMO seeds in Chile is strictly regulated in order to safeguard the environment, maintain biodiversity, and facilitating trade and technological development in agriculture. As in all countries, GMO production in Chile must be pursuant at all times with all country-specific regulations regarding biosafety and be conducted only within the confines of an appropriate regulatory framework.

SAG oversees the importation and planting, under stringent biosafety measures, of GMOs according to Resolution No. 1,523 of 2001. Biosafety refers to the actions or measures applied to a specific GMO, based on its reproductive biology and fitness, to avoid or minimize potential risks in the environment.^15^ Examples of such measures include the type of transport and storage, packaging, labeling, spatial and/or temporary isolation to avoid cross-pollination with sexually compatible species, control of voluntary plants, and post-harvest field management, among others.

SAG carries out a mandatory risk assessment for each GMO that is without a record of prior use in the country. This process is performed on a case-by-case basis considering the particular characteristics of the region where plantings are planned. The process also includes the analysis of introduced genes and proteins produced by GMOs, of history of safe use, of donor organism, of tissues and organs in which the introduced genes are expressed, and of genetic stability. From an agronomic perspective, GMOs are compared with their conventional counterparts, considering morphological, botanical and physiological aspects, potential to survive as a weed, production and viability of pollen, and the possibility of gene transfer to wild plants. Potential effects on non-target organisms and protein degradation in soils are also analyzed.

Applicants must follow a series of steps. A formal request must be submitted describing the objective, species and genetic modification at stake, and the location for seed storage, which must be previously approved by SAG. Once SAG approves an application, it issues a resolution describing all biosafety measures to be met for each case. From import to export or destruction of GMO, SAG will maintain complete traceability of the material. In addition to environmental approval, through another regulatory process and based on the stage of development of a GMO and whether it has had previous authorizations in the country, SAG oversees and authorizes the import of GMOs. When permits are granted, they are only valid for one calendar year.

All farms growing GM seed fields are surveyed by SAG officers throughout the growing season. These officers verify the proper management of GMO seed fields throughout planting, harvesting, processing, and exporting stages. Machinery cleaning protocols are also assessed to prevent the inadvertent propagation of GMOs. SAG has implemented diverse detection methods to confirm the identity of authorized GMO. Immunological strip tests are used to detect specific proteins, and real-time PCR tests are used to detect genes or genetic elements which can be found in GMOs, such as promoters and terminators. To keep full traceability of GMO seeds, all byproducts, waste, and remnants must be destroyed at locations authorized by SAG.

Despite the relevance of the Chilean GM seed industry, local farmers are not authorized to use GMO seeds for farming purposes. Farmers are unable to opt for GMO crops, despite the fact that the environmental law enacted in 2010 permits their use if an environmental risk assessment has been performed. Although the official rules for Environmental Impact Assessments were issued in Chile in 2013, no specific procedures regarding GMOs have been published, causing a regulatory void for the use of GMOs in Chile.

## Coexistence of Organic Farming and GM Seed Industries

It is worth noting that countries with the largest areas devoted to organic agriculture in 2019 were simultaneously the highest producers of GMOs (Table 1). In this context, organic land represented 72.3 million hectares worldwide in 2019, comprising permanent grassland (68%), arable land (18%), permanent crops (6.5%), and others (6%). Thus, Australia, the country with the largest land area devoted to organic agriculture, devoted 97% of its organic lands as grazing areas.^17^ On the other hand, adoption of GM farming practices reached 190.4 million hectares worldwide in 2019. Soybean was the leading GMO with 91.9 million hectares (48% of the global biotechnology crop area) devoted to its cultivation, followed by maize, cotton, and canola (60.9, 25.7, and 10.1 million ha, respectively). Other commercially available GMOs, grown over considerably less extensive land areas, include alfalfa, sugar beets, sugarcane, papaya, safflower, potatoes, eggplants, squash, apples, and pineapples.^16^

**Table 1.** Area of crops (million hectares) grown under organic standards and GMOs in selected countries in 2019.^16,17^

Country	Organic farming	Rank	GMO	Rank
Australia	35.69	1	0.6	13
Argentina	3.67	2	24.0	3
Spain	2.35	3	0.1	17
USA	2.33	4	71.5	1
India	2.30	5	11.9	5
China	2.22	7	3.2	7
Canada	1.32	11	12.5	4
Brazil	1.28	12	52.8	2

To date, no reports of economic ramifications from the unintended presence of GMOs in non-GMO products, including organic products, have been formally issued in Chile. Nonetheless, under normal agriculture conditions, the possibility of adventitious presence of GMOs used by the Chilean GM seed industry in non-GM crops cannot be excluded. Among some of the factors affecting the likelihood of pollen and/or seed transfer between farms, proximity and the mode of transfer stand out. Regarding the physical distance between farms, buffer zones between GMO and non-GMO lands have been found to be capable of very effectively reducing risk.^18^ On the other hand, wind may transfer GM pollen, pesticides, or even smoke – thereby affecting the flavor of products – onto adjacent lands, and irrigation water could transfer pesticides and plant propagation materials. Also necessary to consider are involuntary transfers via humans, animals, vehicles, soil, and admixture (i.e., grain movement), among others. Thus, controlling the movement of farm animals, thorough cleaning of equipment, and meeting the ‘best practices’ management and quality assurance standards are crucial to minimizing the risk of adventitious presence.

## Adventitious Presence Complaints

Some online reports have claimed that Chilean conventional seed shipments were contaminated with GMOs. In 2001, the farm ministry of the northern state of Schleswig-Holstein, Germany, stated that GM maize seed mixed with normal seeds imported from Chile and Canada had been discovered (https://www.gmwatch.org/en/news/archive/2001/8121-German-state-finds-gm-material-in-maize-seed). In 2013, also in Schleswig-Holstein, claims of adventitious presence in corn seed shipment originating from Chile were published. According to the news story, a high probability was found that the GM content of the batch was lower than 0.1%. Due to zero-tolerance policies, which dictates that a seed lot must not contain any rate of GMOs, it was voluntarily withdrawn from the market by the supplier (https://www.proplanta.de/agrar-nachrichten/pflanze/maissaatgut-positiv-auf-gentechnische-verunreinigungen-getestet_article1367519997.html). In both cases, Chilean authorities stated that no formal notification by any official German regulatory agency or organism was received; therefore, such claims have been dismissed due to lack of evidence.

In 2012, the European Union (EU) Food and Veterinary Office conducted an audit to evaluate the procedures currently in place for GMOs regarding seed intended for exportation to the EU. It also aimed to evaluate these procedures in order to prevent adventitious presence in non-GMO seeds exported from Chile to the EU. Given that seeds can first be transported to various countries to be processed before reaching their intended destination, the audit was conducted because competent authorities in the EU had detected traces of GMOs in non-GM maize seed originating from Chile.

Among the conclusions reached by these audits were that the implementation of official control measures for GM seed at the importation and field production phases should ensure, in most cases, that non-GM seed exported to the EU lacks adventious presence of GM seeds. However, the conclusions also highlighted that no official tests are performed that target GMO contamination of non-GM seed intended for exportation to the EU (European Commission, ^19^2012).

In 2008, the detection of GMOs in conventional corn fields within a specific farming region was claimed by the Chilean NGO Sustainable Societies Foundation and notified to SAG. This NGO collected 30 samples of corn in farms located near GM seed fields, four of which were claimed to have tested positive for the presence of GMOs. In this case, through a formal letter accessed by the Chilean Transparency Law, SAG officially stated that the sampling and detection methods used were not suitable, and that the complaint failed to provide information about the number of traces detected. According to SAG, although information was requested to the NGO, it was never provided.

In the EU, the Rapid Alert System for Food and Feed (RASFF) (https://ec.europa.eu/food/safety/rasff_en) is a tool employed by its constituent states to notify of any food safety-related measures and includes a description of all cases related to the detection of unauthorized GMOs in food and feed in the EU. Between 2002–2021, there have been 720 notifications of detection of unauthorized GMOs. Based on the description of these notifications, fewer than 30% of these cases may have been linked to viable seeds, while the remaining cases were related to ingredients and products containing unauthorized GMOs. The majority of these products were originated from China (283), the United States (207), and Canada (82). Other important countries producing GMOs have much fewer notifications and include Argentina (9), Colombia (6), India (5), and Brazil (1). To date, Chile has zero notifications reporting the detection of unauthorized GMOs.

## Current Practices

In Chile, organic farming and GM seed production industries are focused on different kind of crops and vegetables, and have been able to establish a successful, effective coexistence. For instance, whereas GM seed producers primarily grow maize, soybean, or canola, Chilean organic farming primarily grow fruits and vegetables, which eliminates the likelihood of cross-fertilization of GMOs and organic crops due to pollen flow between neighboring fields. In some cases, however, organic farmers grow certain species in much smaller that their GM seed-producing counterparts.

In the case of maize, which is the main seed produced and exported from Chile, during the 2018/2019 season, the highest number of organic maize fields (25) was reported over the last five seasons (Table 2), whereas the largest area covered (65.7 ha) was reached during the 2017/2018 season. These numbers contrast sharply with those calculated from GM maize seed production, as every season there are more than 1,000 locations producing GM maize seed, encompassing thousands of hectares (Table 2). These locations involve a multitude of local and international companies and individual farmers working as growers or seed tollers, thereby creating a broad agricultural network producing high-value seeds.

**Table 2.** Comparison of the number of fields and hectares (in round brackets) of maize devoted to organic and GM production throughout the last 5 seasons in Chile. Based on official data provided by SAG.* N/A: No information available

	2015–2016		2016–2017		2017–2018		2018–2019		2019–2020	
Region	Organic	GMO	Organic	GMO	Organic	GMO	Organic	GMO	Organic	GMO
Arica y Parinacota		115		157		270		301	N/A	328
		(47.5 ha)		(49 ha)		(64 ha)		(45.1 ha)		(49.1 ha)
Coquimbo	2		4		5		2		N/A	
	(0.53 ha)		(11.8 ha)		(3.05 ha)		(1 ha)			
Valparaíso	1	20	2	8	2	14		10	N/A	3
	(8.1 ha)	(25.7 ha)	(1.45 ha)	(14.4 ha)	(8.3 ha)	(18.6 ha)		(13.3 ha)		(5 ha)
Metropolitana	2	471	7	444	8	564	9	399	N/A	311
	(12.9 ha)	(970.9 ha)	(7.21 ha)	(822 ha)	(10.5 ha)	(1,619.4 ha)	(15.5 ha)	(1,293.3 ha)		(786.1 ha)
O`Higgins		163		203	1	211	1	195	N/A	192
		(1,553.7 ha)		(1,597.8 ha)	(0.6 ha)	(2,182 ha)	(1.5 ha)	(1,426 ha)		(1,675.7 ha)
Maule	2	268	3	357	2	332	3	280	N/A	437
	(15.48 ha)	(2,072.8 ha)	(2.5 ha)	(2,900 ha)	(2.65 ha)	(3,936.2 ha)	(4.5 ha)	(2,639.8 ha)		(5,728.2 ha)
Ñuble	2	1	3		4	1	6	1	N/A	2
	(0.5 ha)	(11 ha)	(24.82 ha)		(39.8 ha)	(2 ha)	(41 ha)	(9.9 ha)		(41.2 ha)
Araucanía	1		1		1		1		N/A	
	(0.5 ha)		(1.5 ha)		(0.5 ha)		(0.25 ha)			
Los Lagos			1		1		3		N/A	
			(1 ha)		(0.3 ha)		(0.03 ha)			
Number of fields	10	1038	21	1169	24	1392	25	1186	N/A	1273
Hectares	(38.01 ha)	(4,681.6 ha)	(50.28 ha)	(5,383.2 ha)	(65.7 ha)	(7,822.2 ha)	(63.78 ha)	(5,427.4 ha)	N/A	(8,285.3 ha)

It is worth noting that not all GMO maize seed production in Chile is conducted by private companies; designated universities and other academic organizations also produce GM seeds for research purposes. Farmers who produce non-GM crops must meet tolerance levels for GM adventitious presence imposed by local and foreign buyers and by other countries. If these are unmet, farmers may lose their status as organic producers and may be left to assume all transportation and marketing costs required to sell the crop within alternative markets.^20^ In this context, according to the EC Organic Regulation (Article 9.2 of Council Regulation (EC) No. 834/2007 and Article 11.2 of Regulation (EU) 2018/848), products containing traces of GMOs may be labeled as organic products, unless the general labeling thresholds are exceeded. Thus, products containing GMOs up to 0.9% per ingredient or component may still be marketed as organic products, if this proportion is accidental or technically unavoidable. However, the aim must be to limit the occurrence of GMOs in organic products below GMO labeling requirements to the lowest possible level.^21^

In the United States, the National Organic Program policy states that trace amounts of GMOs does not automatically identify the farm as being in violation of the USDA regulations for organic products. In such instances, the certifying agent will examine how the adventitious presence occurred and recommend interventions to prevent its future occurrence (e.g., larger buffer zones, more thorough cleaning of a shared grain mill).^22^ To this end, it has been proposed that the outcrossing potential for GM maize and cultivated relatives in Chile can be reduced if efficient reproductive isolation is implemented. A multi-tiered approach is proposed that encompasses isolation distances, buffer zones, discard zones, and scattered sowing dates.^23^

Although field situations across various regions or countries may differ in extension, pollen sources, flowering time, and weather, a science-based isolation distance of 40 m has been proposed for European agriculture to avoid admixture in maize cultivation exceeding a threshold of 0.9%.^24^ The same isolation distance was observed in Uruguay, which was the shortest tested, and was sufficient to retain the threshold below 0.9%.^25^

On the other hand, there are no official records of canola grown through organic practices in Chile (Table 3). Perhaps this is because the yield of oilseed production is low and variable in organic farming of this crop. Weed pressure and lack of tools for efficient weed control (e.g., synthetic herbicides are not permitted during organic farming) are the main factors impairing organic canola production.^26^

**Table 3.** Comparison of the number of fields and hectares (in round brackets) of canola devoted to organic and GM production throughout the last 5 seasons in Chile. Based on official data provided by SAG. *N/A: No information available

	2015–2016		2016–2017		2017–2018		2018–2019		2019–2020	
Región	Organic	GMO	Organic	GMO	Organic	GMO	Organic	GMO	Organic	GMO
Arica y Parinacota		8		10		8		10	N/A	10
		(0.31 ha)		(3.31 ha)		(2.50 ha)		(1.4 ha)		(1.3 ha)
Metropolitana		14		11		12		11	N/A	16
		(19.01 ha)		(16.69 ha)		(13.96 ha)		(15.1 ha)		(8.6 ha)
O`Higgins		7		9		13		7	N/A	10
		(3.88 ha)		(4.59 ha)		(8.98 ha)		(7.3 ha)		(13.2 ha)
Maule		29		26		42		68	N/A	51
		(706.4 ha)		(632.32 ha)		(795.12 ha)		(1,026.3 ha)		(837.1 ha)
Ñuble		47		29		51		45	N/A	60
		(948.34 ha)		(525.2 ha)		(1,142.1 ha)		(876.1 ha)		(764.2 ha)
Biobío		37		25		31		23	N/A	28
		(792.81 ha)		(576.51 ha)		(952.75 ha)		(759.1 ha)		(745.2 ha)
Araucanía		68		67		85		93	N/A	66
		(492.66 ha)		(370.74 ha)		(784.03 ha)		(810 ha)		(509.8 ha)
Number of fields	0	210	0	177	0	242	0	257	N/A	241
Hectares	(0 ha)	(2,960.43 ha)	(0 ha)	(2,129.36 ha)	(0 ha)	(3,699.44 ha)	(0 ha)	(3,495.3 ha)	N/A	(2,879.4 ha)

In soybeans, the largest area of land dedicated to organic farming was 2.8 ha in the 2018/2019 season, which contrasts strikingly with the 1,500–3,000 ha dedicated to GM soybean seed production every season in Chile (Table 4). Furthermore, soybean is a largely self-pollinating crop; thus, the likelihood of cross pollination is negligible.^23^ It is worth noting that the soybean is not an attractive crop for Chilean farmers, because it requires a minimum of 300 mm of water throughout the productive season, which in Chile is provided by irrigation. Irrigation renders competition for Chilean farmers against soybean imports from Argentina, Brazil, Paraguay, and Uruguay prohibitively expensive. In those countries, the management of soybean cultivation is highly efficient, with higher yields and lower production costs.^27^

**Table 4.** Comparison of the number of fields and hectares (in round brackets) of soybean devoted to organic and GM production throughout the last 5 seasons in Chile. Based on official data provided by SAG. *N/A: No information available

	2015–2016		2016–2017		2017–2018		2018–2019		2019–2020	
Region	Organic	GMO	Organic	GMO	Organic	GMO	Organic	GMO	Organic	GMO
Arica y Parinacota		16		14		9		6	N/A	4
		(1.5 ha)		(0.26 ha)		(0.5 ha)		(0.14 ha)		(1 ha)
Coquimbo					1		1		N/A	
					(0.35 ha)		(1 ha)			
Valparaíso		7		13		14		4	N/A	4
		(15 ha)		(39.4 ha)		(46.9 ha)		(1.8 ha)		(2.6 ha)
Metropolitana		28		64		56	1	20	N/A	18
		(125.7 ha)		(308.2 ha)		(251.8 ha)	(0.3 ha)	(42.3 ha)		(155.5 ha)
O`Higgins		182		134		92		129	N/A	96
		(457.3 ha)		(735.9 ha)		(840.5 ha)		(702 ha)		(841 ha)
Maule		110		98		138		91	N/A	154
		(953.6 ha)		(1,206.4 ha)		(1,165.4 ha)		(950.1 ha)		(1,828.5 ha)
Ñuble		17		32		17		28	N/A	60
		(109 ha)		(259.36 ha)		(103.4 ha)		(108.2 ha)		(289.5 ha)
Biobío	1		1	2	1		1	1	N/A	7
	(2.5 ha)		(0.5 ha)	(62.85 ha)	(0.5 ha)		(1 ha)	(0.04 ha)		(51.5 ha)
Araucanía		1	1	3	1		1		N/A	
		(0,1 ha)	(0,5 ha)	(45,7 ha)	(0,01 ha)		(0,5 ha)			
Number of fields	1	361	2	360	3	326	4	279	N/A	343
Hectares	(2.5 ha)	(1,662.2 ha)	(1 ha)	(2,658.02 ha)	(0.86 ha)	(2,408.5 ha)	(2.8 ha)	(1,804.58 ha)	N/A	(3,169.6 ha)

With respect to certified organic honey, only a small number of beekeepers are registered annually (Table 5). For instance, in 2019, only 10 producers were registered, three of whom were located in the Los Lagos region, where there is no production of GMOs. In previous years, organic honey was also produced in the Coquimbo and Los Ríos regions, both of which also did not produce GMOs due to unfavorable weather conditions.

**Table 5.** Number of certified organic beekeepers in Chile by year and region. Data are based on official data provided by SAG

	Year				
Region	2015	2016	2017	2018	2019
Coquimbo	1			1	
Valparaíso	1		2	3	
Metropolitana	3	4	4	9	1
O`Higgins		10	8	12	4
Maule	1		3	4	
Ñuble		2	1	1	1
Biobío				1	1
Araucanía	1	1	1	2	
Los Ríos			1	2	
Los Lagos	3	5	9	17	3
	10	22	29	52	10

Beekeepers seeking organic certification typically operate in locations far from activities that may threaten their certification. Furthermore, it is worth noting that conventional, non-GM crops are not suitable forage for bees for the purpose of producing organic honey. Consequently, growing GM crops for seed production in locations regularly used for conventional farming would have no additional effect on organic honey production. Moreover, SAG has implemented the “National Apicultural Consultation Geographic System,” which allows beekeepers to install and relocate their hives in locations free of GMOs (https://www.sag.gob.cl/ambitos-de-accion/sig-apicola). All beekeepers included in the Registry of Export Honey Beekeepers, which is an official requirement for exporting honey, can access such system. When entering GPS coordinates of an apiary on the software, a report is generated, providing information about the location of GMO seed fields covering a radius of 5 km, which is known as the GMO influence area, or 10 km, which is known as the GMO preventing influence area. Also, the report indicates the species, field extension, sowing dates, and the approximate dates of the onset and termination of the flowering of the GMO. Therefore, there are no practical incompatibilities between the production of high-quality organic honey and of GM seeds in Chile.

## Conclusions

At present, both organic farmers and producers of GM seeds are effectively coexisting in Chile. The direct impacts of GM seeds of maize, canola, and soybean on organic production in Chile are likely to be negligible, and not a single case of agronomic, quality, or commercial impact has been reported, notified, and confirmed to date. Under Chilean organic certification standards, organic production is to be isolated from the production of any non-organic products. Organic farmers are already required to establish stringent measures to avoid contact with non-organic crops, regardless of whether these crops are conventional or GM. The successful coexistence between organic agriculture and GMO production is not only feasible in Chile, it has already taken place over the past several years, providing farmers with additional options and alternatives. The Chilean experience to date on the coexistence described generates valuable lessons that may prove valuable to other countries – including developing countries – considering GM crops as part of the production options available to farmers. Regarding the current situation of both activities, Chile’s GMO seed industry is unlikely to have any deleterious impact on the organic food production sector.
